# Supplementary material for: MetaRibo-Seq measures translation in microbiomes
Source: Nat Commun. 2020 Jun 29;11:3268. doi: 10.1038/s41467-020-17081-z (PMC7324362; doi:10.1038/s41467-020-17081-z)
Supplement: Supplementary file 10 — Supplementary Data 7 [file 41467_2020_17081_MOESM10_ESM.zip › File2/Confidence_High_Taxonomy/61675_out.krona.html]

Javascript must be enabled to view this page.

members
magnitude
magnitudeUnassigned
count
unassigned
taxon
rank

61675\_out

17

2
17
superkingdom

phylum
1
976

class
1
200643

order
171549
1

1
815
family

genus
1
816

1
817
species

SRS147139\_contig\_number\_31432

16
1239
phylum

16
186801
class

16
186802
order

family
186803
16

genus
1

SRS063040\_contig\_number\_13910
841
2

species

SRS016095\_contig\_number\_13981
1
360807


SRS015782\_contig\_number\_31405SRS053335\_contig\_number\_14733SRS147766\_contig\_number\_9955
6
572511
genus
3

species

SRS077086\_contig\_number\_contig-100\_1891.72609
1
1896976

2
2292958
species

SRS144714\_contig\_number\_10302SRS146813\_contig\_number\_29431

genus
8
1407607

species

SRS015578\_contig\_number\_21349SRS016335\_contig\_number\_6814SRS018936\_contig\_number\_3049SRS075878\_contig\_number\_22541SRS104400\_contig\_number\_contig-100\_1213.342247SRS144537\_contig\_number\_26698SRS147919\_contig\_number\_19143SRS149879\_contig\_number\_13172
8
1150298
